# Anthocyanin Profiles in Flowers of Grape Hyacinth

**DOI:** 10.3390/molecules22050688

**Published:** 2017-04-26

**Authors:** Qian Lou, Lin Wang, Hongli Liu, Yali Liu

**Affiliations:** 1 College of Horticulture, Northwest A & F University, Yangling 712100, Shaanxi, China; louqian@nwsuaf.edu.cn; 2 Key Laboratory of Biology and Genetic Improvement of Horticultural Crops (Northwest Region), Ministry of Agriculture, Yangling 712100, Shaanxi, China; lynnw7@163.com (L.W.); liuhongli1221@sina.com (H.L.); 3 State Key Laboratory of Crop Stress Biology in Arid Areas, Northwest A&F University, Yangling 712100, Shaanxi, China; 4 College of Landscape Architecture and Arts, Northwest A & F University, Yangling 712100, Shaanxi, China

**Keywords:** anthocyanin, acylation, flower color, methylation, *Muscari*

## Abstract

Grape hyacinth (*Muscari* spp.) is a popular ornamental bulbous perennial famous for its blue flowers. To understand the chemical basis of the rich blue colors in this plant, anthocyanin profiles of six blue flowering grape hyacinths as well as one pink and one white cultivar were determined using high-performance liquid chromatography and mass spectrometry. Along with two known compounds, eight putative anthocyanins were identified in the tepals of grape hyacinth for the first time. The accumulation and distribution of anthocyanins in the plant showed significant cultivar and flower development specificity. Violet-blue flowers mainly contained simple delphinidin-type anthocyanins bearing one or two methyl-groups but no acyl groups, whereas white and pink flowers synthesised more complex pelargonidin/cyanidin-derivatives with acyl-moieties but no methyl-groups. The results partially reveal why solid blue, orange or red flowers are rare in this plant in nature. In addition, pelargonidin-type anthocyanins were found for the first time in the genus, bringing more opportunities in terms of breeding of flower color in grape hyacinth.

## 1. Introduction

Grape hyacinth (*Muscari* spp.) is an ornamental bulbous flower known for its kaleidoscopic palette of blue color—azure, cobalt, violet, lavender, or purple. They are perfect for rock gardens, borders, or as ground cover, adding rare “cool” colors to a spring garden. It is an ideal plant to study the mechanism of blue flower formation.

In nature, blue colors in higher plants are derived from anthocyanin [1]. It is a common water-soluble pigment and further separated into three types: pelargonidin (Pg), cyanidin (Cy), and delphinidin (Dp), with different B-ring hydroxylation patterns [2]. The more hydroxyl-groups on the B-ring, the greater the anthocyanin hue shifts to blue [3]. Pg contains a hydroxyl-group and produces orange to pink to red colors. Cy has two hydroxyl-groups and shows a characteristic reddish-purple color. Dp, with three hydroxyl-groups tends to give blue hues to flowers [2]. Besides the correct pigments (usually Dp), the occurrence of a blue flower also requires a series of complex mechanisms, such as vacuolar alkalization, polyacylation modification, metal complexation and/or co-pigmentation [1]. The blue coloration of Japanese morning glory is achieved by vacuolar alkalization. As flowers open, their colour changes from reddish-purple to pure blue with a rise of vacuoles pH [4]. The ternatins of butterfly peas and the viodelphin/cyanodelphin of delphinium demonstrate the importance of polyacylation on blue color formation [2]. Studies on tulips have shown that the color blue is achieved by having the correct pigments in conjunction with certain Fe^2+^ ions [5]. Thus, we have reason to believe that the blue coloration mechanism of different plants is rich and varied. This situation increases the difficulty of blue flower molecular breeding. To solve the problem, accumulation of biochemical information on blue flower pigments is one of the indispensable requisites.

In *Muscari*, Mori et al. [6] determined the anthocyanins of 13 different species by high-performance liquid chromatography (HPLC) and found that blue flowering grape hyacinth mostly accumulated Dp, but flowers with lilac or reddish purple colors accumulated different proportions of Dp and Cy. No anthocyanins were detected in white flowering grape hyacinth (*M. botryoides* ‘Album’). When the synthesis pathway of Dp and Cy was blocked, the flowers of *M. armeniacum* could not accumulate blue pigments, making them white in color [7]. In addition, Yoshida et al. [8] isolated and structurally identified muscarinin A, an acylation anthocyanin, from *M. armeniacum*. There have been no reports concerning the existence of Pg-type anthocyanins in the genera *Muscari* until now. This might be the root cause of rare orange-to-pink-to-red flowers in grape hyacinth. In 2011, a pale pink flowering *Muscari* ‘*Pink Surprise*’ plant was first described. It had a previously undiscovered color in the genus [9], which provided a new perspective to discovering the color secrets of grape hyacinth.

Here, one pink, one white, and six blue grape hyacinth cultivars were used as materials to analyse the composition of pigments during flower development. We found the reason for pink flowers and the importance of methylation and acylation in flower color development in the genus.

## 2. Results

### 2.1. Anthocyanin Components in Grape Hyacinth

**Figure 1:**
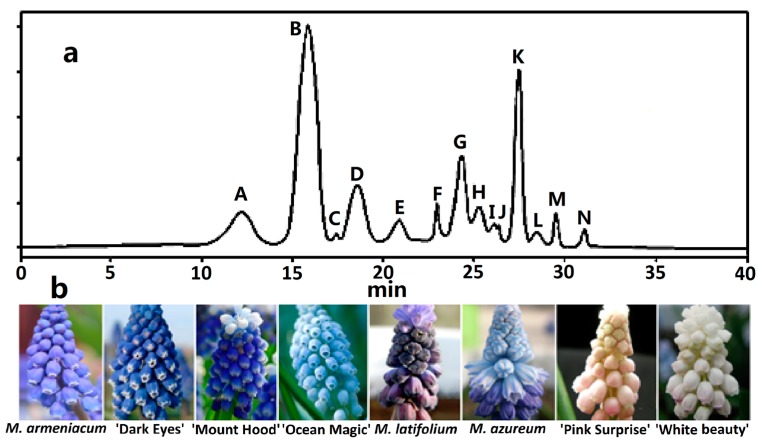
HPLC chromatogram of anthocyanin profiles in eight grape hyacinth cultivars. (**a**) Schematic diagram of retention time of anthocyanins in grape hyacinth tepals. Refer to Table 1; (**b**) Eight grape hyacinth cultivars from left to right: *M. armeniacum*, *M. armeniacum* ‘Dark Eyes’, *M. aucheri* ‘Mount Hood’, *M. aucheri* ‘Ocean Magic’, *M. latifolium*, *M. azureum*, *M.* ‘Pink Surprise’, *M. aucheri* ‘White beauty’.

In total, 14 anthocyanins were identified in the tepals of grape hyacinth (Figure 1, Figure S1 and Table 1). Peaks A and G, previously found in grape hyacinth, were confirmed by the result of MS (Mass Spectrometry) [6,7,10]. Peak A had a molecular ion at mass-to-charge ratio (*m*/*z*) 465 and two mass fragment ions, one at *m*/*z* 303, due to the elimination of one molecule of Dp, and one at *m*/*z* 162 due to the loss of glucose. Therefore, the peak was identified as delphinidin-3-*O*-glucoside (Dp3G). Peak G showed molecular cations at *m*/*z* 287, pointing to Cy. Peaks B, D, F, H, I, J, K, and L were identified in this plant for the first time. Similarly, peaks B and D had molecular and fragment cations of *m*/*z* 479 and 317 as well as 493 and 331, identified as petunidin-3-*O*-glucoside (Pt3G) and malvidin-3-*O*-glucoside (Mv3G), respectively. Peak F had a molecular ion at *m*/*z* 889 and three mass fragment ions, one at *m*/*z* 757, due to the elimination of one molecule of arabinoside, one at *m*/*z* 595, indicating the loss of caffeoyl-group, and one at *m*/*z* 271 for the loss of sophoroside. Thus, peak F could be confirmed as pelargonidin-3-*O*-caffeoylsophoroside-5-*O*-arabinoside (Pg3CaSop5Ara). Peaks H and I showed a characteristic fragment ion at *m*/*z* 287, pointing to Cy derivatives. Peak H, with molecular and mass ions from MS results of *m*/*z* 757 and 595 (loss of caffeoyl-group), respectively, was identified as cyanidin-3-*O*-caffeoylrutinoside (Cy3CaRu). Peak I showed a molecular cation of *m*/*z* 887 and a fragment ion of *m*/*z* 757 for the loss of malonyl-group, inferred as cyanidin-3-*O*-(*p*-coumaroyl)-glucoside-5-*O*-malonylglucoside (Cy3pCG5MaG) according to the anthocyanin composition in the allied species of the Hyacinthaceae (Figure S2) [11,12]. Peaks J, K, and L were assigned as Pg derivatives (*m*/*z* 271). Peak J had a molecular ion at *m*/*z* 801 and three mass fragment ions, one at *m*/*z* 595, due to the elimination of one molecule of sinapylglucoside, one at *m*/*z* 433, indicating the loss of sinapylglucoside, and one at *m*/*z* 271 for the loss of sophoroside. Thus, peak J could be confirmed as pelargonidin-3-*O*-sinapylglucoside-5-*O*-glucoside (Pg3SiG5G). Peak K showed a molecular cation of *m*/*z* 771 as well as fragment ions of *m*/*z* 565 (loss of sinapyl) and *m/z* 433 (loss of arabinoside), identified as pelargonidin-3-*O*-sinapylglucoside-5-*O*-arabinoside (Pg3SiG5Ara). Peak L showed a molecular ion at *m/z* 741 as well as mass fragment ions at *m*/*z* 565 (loss of ferulyl-group) and *m*/*z* 433 (loss of arabinoside). Thus, peak L could be confirmed as pelargonidin-3-*O*-ferulyl-glucoside-5-*O*-arabinoside (Pg3FeG5Ara). It is worth noting that no acylated Dp derivative was detected in all the grape hyacinth tepals in this study. Previously, two acylation Dp derivatives, Dp-3-(6-*p*-coumaroylglucoside)-5-(4-rhamnosyl-6-malonylglucoside) (muscarinin A), were isolated from *M. armeniacum* [8]. The molecular weight of muscarinin A was 1005 [8]. Here, although we detected few fragment cations of *m*/*z* 1005 and 859, their retention time did not correspond with the chromatographic peak. In addition, the fragments did not completely break into specific aglycone fragments, so we ignored the information in later analysis. Moreover, because the structure of some anthocyanins was only presumed from MS data, further study will be necessary to isolate and identify the structure of these anthocyanins.

### 2.2. Specific Accumulation and Distribution of Anthocyanins in Grape Hyacinth

Flower development of grape hyacinth was divided into five stages: stage 1—closed buds with white or green tepal; stage 2—closed buds, tepal pigmentation starts; stage 3—closed buds just before blooming; stage 4—opening of flowers; and stage 5—flowers during senescence (Figure S3). In most cultivars, total anthocyanins (TA) increased with the development of flowers and reached peak values just before the buds opened, after which the TA degraded gradually or decreased as a result of petal expansion (Figure S2, Clade 2). Unlike the others, a steep rise in TA of *M. armeniacum* occurred and subsequently peaked at stage 5 (Figure S2, Clade 1). At the same time, in ‘Pink Surprise’ and ‘White Magic’, the TA content was higher in young buds, which is similar to that of *Eustoma grandiflorum* in terms of protection from ultraviolet radiation (Figure S2, Clade 4) [13].

**Figure 2:**
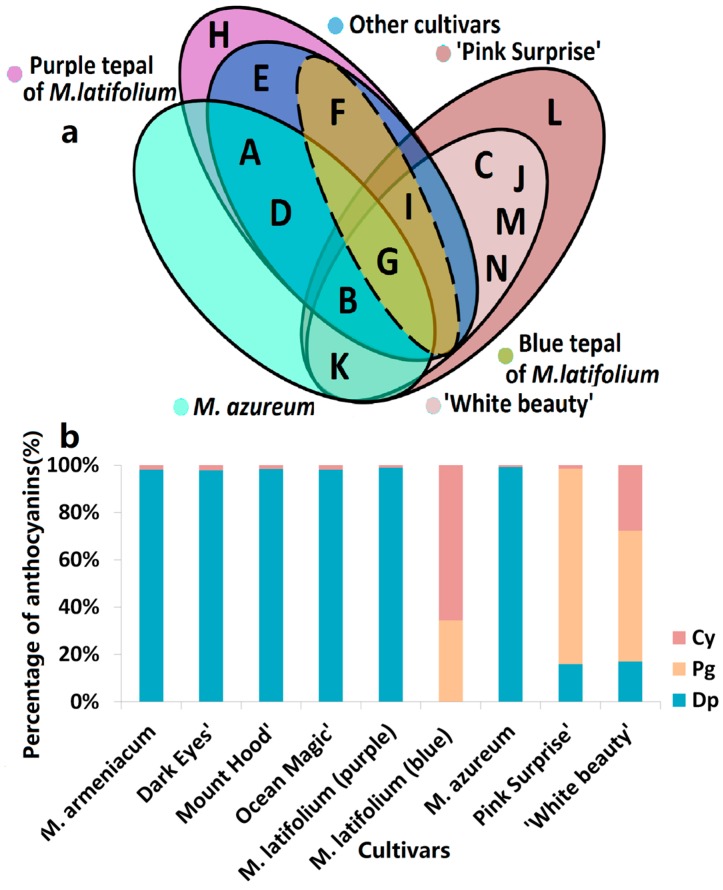
Anthocyanins distribution in tepals of eight grape hyacinth cultivars. (**a**) Venn diagram of anthocyanins distribution in different grape hyacinth cultivars. A–K: Refer to Table 1; (**b**) Percentage of Dp, Cy, Pg-type anthocyanins in tepals of different grape hyacinth cultivars. Flower color was recorded as three-dimensional CIEL*a*b* values and reproduced by photoshop using L, a*, b* values at stage 4. Cy—cyanidin; Dp—delphinidin; Pg—pelargonidin.

**Figure 3:**
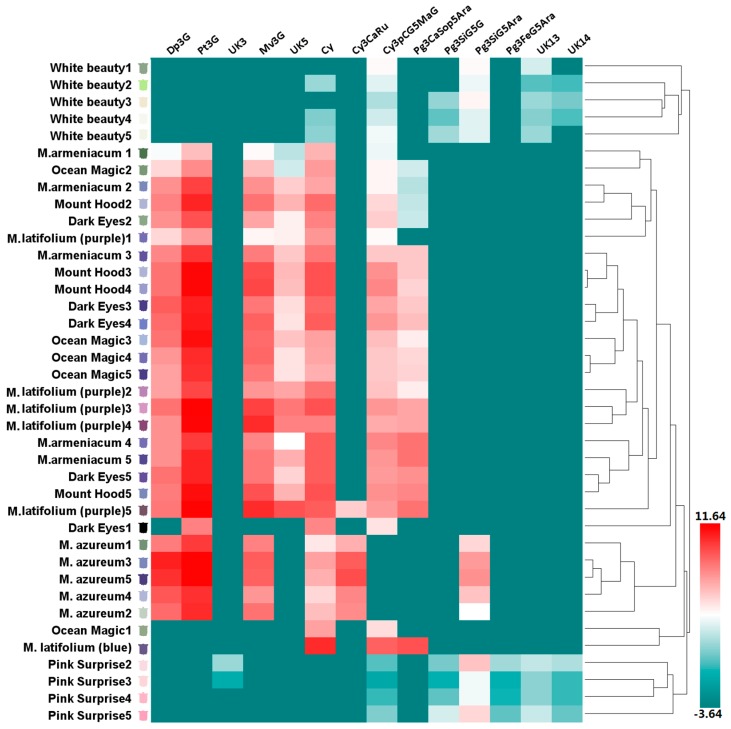
Anthocyanin profiles in tepals of eight grape hyacinth cultivars. The *Y*-axis represents tepals at stages 1–5 from different grape hyacinth cultivars. The X-axis represents the relative content of individual anthocyanins in different tepals. The average anthocyanin content of three independent replicates was shown on grids with different color scale levels representing the relative log2 at different samples, respectively. Flower color was recorded as three-dimensional CIEL*a*b* values and reproduced by photoshop using L, a*, b* values at all development stages investigated in this study. Cy—cyanidin; Cy3CaRu—cyanidin-3-*O*-caffeoyl-rutinoside; Cy3pCG5MaG—cyanidin-3-*O*-(*p*-coumaroyl)-glucoside-5-*O*-malonyl-glucoside; Dp3G—delphinidin-3-O-glucoside; Dp3G—delphinidin-3-*O*-glucoside; Mv3G—malvidin-3-*O*-glucoside; Pg3CaSop5Ara—pelargonidin-3-*O*-caffeoyl-sophoroside-5-*O*-arabinoside; Pg3FeG5Ara—pelargonidin-3-*O*-ferulylglucoside-5-*O*-arabinoside; Pg3SiG5Ara—pelargonidin-3-*O*-sinapyl-glucoside-5-*O*-arabinoside; Pg3SiG5G—pelargonidin-3-*O*-sinapyl-glucoside-5-*O*-glucoside; Pt3G—petunidin-3-*O*-glucoside; UK—unknown compound.

According to the color of opening flowers, eight grape hyacinth cultivars were divided into four groups: white (including ‘White Beauty’), violet-blue (including *Muscari armeniacum*, ‘Dark Eyes’, ‘Mount Hood’, ‘Ocean Magic’, *M. azureum*, and upper flower of *M. latifolium*), purple (including the lower flower of *M. latifolium*) and red-purple (including ‘Pink Surprise’; Table S1). Anthocyanin composition differed strongly among cultivars in different groups (Figure 2). On the basis of clustering and distribution analysis of anthocyanin profiles, white and red-purple cultivars were gathered into different clusters, respectively (Figure 3, Table S2). Pg3FeG5Ara was unique to red-purple groups (Figure 2). Besides that, the anthocyanins in red-purple and white groups were the same in this type but different in their content and proportion (Figure 2). The main pigment of pink flowers was Pg3SiG5Ara, while those of white flowers were Pg3SiG5Ara and Cy3pCG5MaG. Violet-blue and purple cultivars gathered into another cluster (Figure 3, Table S2). They mainly accumulated Dp-type anthocyanins (Figure 2), especially Pt3G, Mv3G, and Dp3G (Figure 3, Table S1). In fully pigmented tepals, the relative contents of these compounds were 54%, 16%, and 10%, on average, respectively. However, the opposite situation occurred in *M. latifolium*, which belongs to a different sub-cluster from other blue flowers (Figure 3, Table S1). It produced violet-blue tepals via Pg and Cy-type pigments (35.67% and 68.33%, respectively; Figure 2) rather than Dp-type pigments. In addition, *M. azureum* also belongs to an independent sub-cluster (Figure 3, Table S1). It might be due to the white stripe in blue petals or more distant relationship with other cultivars. Then, modifications to anthocyanins in all cultivars were analysed. Interestingly, the violet-blue groups mainly synthesised very simple anthocyanins bearing one or two methyl-groups and a single sugar, whereas the white and red-purple groups accumulated more complex anthocyanins with one or two sugars and acyl-moieties but no methyl-groups (Figure S4).

## 3. Discussion

Grape hyacinths are famous for their blue flowers. However, not all blues in these flowers are true blue. In this study, the so-called blue flowers were violet-blue, purple-violet, or purple (Table S1). Actually, as yet, it is hard to see solid blue as well as orange, pink or red in the genus. This is mainly owing to the large number of Dp derivatives, the small number of Cy derivatives, and few Pg derivatives. Moreover, anthocyanin modification also plays a key role in the final flower colors of grape hyacinth. Two Dp derivatives, Pt3G and Mv3G, are the predominant pigments causing the violet-blue or purple coloration in most grape hyacinth cultivars. Both of them are *O*-methylated anthocyanidins, and the only two *O*-methylated anthocyanidins in all detected cultivars, suggesting the prevalence and specificity of the methylation modification in the genus *Muscari*. Some studies have suggested that methylation gives anthocyanins a slight red hue [14]. Mono- and di-methyl derivatives (Mv3G and peonidin-3-glucoside) contribute a lot to the coloration of red-skinned grapes [15,16]. Mv3G are the most abundant anthocyanin in pink petals of lotus, followed by Dp3G and Pt3G [17,18]. In research on tree peony and paeonia, an increase in the number of methyl-groups in anthocyanidin results in a shift of the color towards purple [19,20]. Thus, methylated Dp-type anthocyanins (Pt3G and Mv3G) might give grape hyacinth a little more purple or red color than unmethylated Dp or Dp3G.

For the white and red-purple group cultivars, Pg and Cy-type anthocyanins are the predominant pigments, and acylation takes the place of methylation to become the predominant modification. Normally, anthocyanins with more aromatic acyl-groups tend to display more blue color than non-acylated anthocyanins [2]. In a great many blue flowers, acylated anthocyanins are the main pigments. The ornamental plants delphiniums and gentians, which are notable for their intense blue color, accumulate polyacylated delphinidin ‘violdelphin’, ‘cyanodelphin’ and ‘gentiodelphin’ in their petals, respectively [21,22,23]. The violet-blue flowers of bellflower [24], poppy anemone [25], and cineraria accumulate a huge amount of Dp derivatives decorated with two or more aromatic residues [26]. In grape hyacinths, acylated anthocyanins comprise 76.67–82.12% of all nine kinds of anthocyanins in white and red-purple group flowers (Figure S3). Interestingly, violet-blue tepals of *M. latifolium* also accumulated huge amounts of Pg and Cy-type derivatives, but no Dp derivatives. In contrast to the previous assumptions based on HPLC [10], further MS results pointed to the importance of acylation in the coloration of these plants. A similar case exists for *Ipomoea purpurea*. Acylated cyanidin glycosides with caffeic acid and/or *p*-coumaric acid rather than Dp-type anthocyanins display a stable violet-blue color [27]. Therefore, we speculate that methylated Dp-type anthocyanins provide the so-called blue flowering grape hyacinth with a red hue (purple or violet-blue), whereas acylation in Cy and Pg-type anthocyanins lead pink flowers to become a bluer color (red-purple). This fact partially explains why solid blue or orange or red flowers are rare in grape hyacinth.

## 4. Materials and Methods

### 4.1. Plant Materials

The eight grape hyacinth cultivars in the study were *M. armeniacum* ‘Dark Eyes’, *M. aucheri* ‘Mount Hood’, *M. aucheri* ‘Ocean Magic’, *M. latifolium*, *M. azureum*, *M.* ‘Pink Surprise’, and *M. aucheri* ‘White beauty’ (Figure 1). All the bulbs were purchased from Zhejiang Hongyue Seeds Company Limited (Zhejiang , China) and planted in an experimental field at the Northwest A&F University (Xi’an, Shaanxi, China). The fresh tepals of each cultivar were sampled in April the following year for color measurement according to the methods described by Qi [10]. All determinations were performed with five replicates. Flowers in some varieties were hard to find in part of the corresponding period during development, including flower buds at stage 1 in *M.* ‘Pink Surprise’ and *M. aucheri* ‘Mount Hood’.

### 4.2. HPLC Analysis

Extraction of anthocyanidins was performed as previously described with a slight modification [28]. The fresh petals were first freeze-dried and ground into fine powder. Then, the resulted flour was macerated with methanol:water:formic acid:TFA (70:27:2:1, *v/v*) and left to rest in the dark at 4 °C for 24 h. The extracted fluid was filtered with 0.22 μm membrane filter and used in subsequent experiments. Hydrolysis analysis of anthocyanins was performed according to Huang [29] and Morit [30]. An aliquot of 300 μL sample concentrations mentioned in HPLC analyses was transferred to a fresh tube, acid-hydrolyzed by adding 300 µL of 6 M HCL, incubated at 90 °C for 1 h. Hydrolyzation solution was immediately cooled to room temperature, and then was filtered prior to injection for analysis.

HPLC analysis used a Waters 600 series high-performance liquid chromatograph (Waters, Milford, MA, USA), a Waters 2487 UV detector (Waters, Milford, MA, USA), an Azbil ADC15 column oven (Azbil, Sanbu, Japan), a Rheodyne 7725i manual injector (Rheodyne of IDEX, Chicago, IL, USA), and a Waters Empower Build 1154-C software (Waters, Milford, MA, USA). An aliquot of 300 µL sample concentrations mentioned in HPLC analyses was transferred to a fresh tube, acid-hydrolyzed by adding 300 μL of 6 M HCL, incubated at 90 °C for 1 h. Hydrolyzation solution was immediately cooled to room temperature, and then was filtered through a 0.22 μm filter membrane prior to injection for analysis. The chromatographic column was a C18 TSK gel ODS-80Ts QA (250 mm × 4.6 mm i.d., 5 μm) (Tosoh, Yokkaichi, Japan), with a flow rate of 0.8 mL/min and injection amount of 10 μL. Mobile phase A employed a 0.1% formic acid solution and phase B an 80% acetonitrile solution. The elution programme was as follows: 0 min, 88% A, 12% B; 15 min, 75% A, 25% B; 32 min, 62% A, 38% B; 40 min, 62% A, 38% B; 45 min, 88% A, 12% B; 50 min, 88% A, 12% B. The detection wavelength was 530 nm. Results of HPLC were verified by at least three independent experiments. Cy, Dp, delphinidin-3-*O*-glucoside (Dp3G), malvidin-3-*O*-glucoside (Mv3G), Pg, petunidin-3-*O*-glucoside (Pt3G) were purchased from ChromaDex (Santa Ana, CA, USA). The concentration of anthocyanidins was quantified by external reference methods using Dp-chloride as standards.

### 4.3. High-Performance Liquid Chromatography–Mass Spectrometry (LC-MS) Analysis

High-performance liquid chromatography–quadrupole time-of-flight mass spectrometry (Waters, Milford, MA, USA) and an Acquity UPLC BEH C18 column (100 mm × 2.1 mm, 1.7 μm) were used for MS analysis. The HPLC procedures included the following: flow rate of 0.3 mL/min; injection amount of 10 µL; and mobile phase A with 0.1% formic acid solution and phase B with 80% acetonitrile solution. The elution programme was as follows: 0 min, 88% A, 12% B; 6 min, 75% A, 25% B; 13 min, 62% A, 38% B; 16 min, 62% A, 38% B; 18 min, 88% A, 12% B; 20 min, 88% A, 12% B. Mass spectrometry (MS) settings included the following: electrospray ionization (ESI); positive ion detection mode; scanning scope of 100–1000 *m*/*z*; capillary voltage of 3000 V; cone voltage of 45 V; extract cone voltage of 4 V; solvent removal of nitrogen flow rate of 40 L/h; ion source temperature of 100 ·C; and drying temperature of 350 ·C.

### 4.4. Statistical Analysis

In order to understand the accumulation patterns of anthocyanins in different samples, the average value of anthocyanin content (*n* = 3) was log2 transformed to enhance the homogeneity of the data and normalised using the computer software HemI 1.0 (The Cuckoo Workgroup, Wuhan, China).

## 5. Conclusions

This study focuses on flower color and anthocyanin profiles of grape hyacinth. In the course of the study, we identified 14 anthocyanins in this plant, which distributed at various amounts in petals of different colors. Among them, methylated Dp-type anthocyanins are the predominant pigments causing the violet-blue or purple coloration. Acylation Cy and Pg-type anthocyanins lead to the red-purple coloration of grape hyacinth flowers. However, the mobilisation of anthocyanin does not seem to fully explain the delicate and varied color in this plant. Further study on co-pigmentation, metal complexation, and vacuolar alkalisation is necessary to understand the mechanism of color formation in *Muscari*.

## Figures and Tables

**Table 1:** Identification of anthocyanins in eight grape hyacinth cultivars.

Peak No. ^a^	Compound	Abbreviations	Retention Time (min)	Molecular Ion (*m*/*z*)	Fragment Ions (*m*/*z*)
A	Delphinidin 3-*O*-glucoside	Dp3G	12.4	465	465/303
B	Petunidin 3-*O*-glucoside	Pt3G	16.2	479	479/317
C	Unknown compound	UK3	17.8	-	-
D	Malvidin 3-*O*-glucoside	Mv3G	18.9	493	493/331
E	Unknown compound	UK5	21.2	-	-
F	Pelargonidin-3-*O*-caffeoylsophoroside-5-*O*-arabinoside	Pg3CaSop5Ara	23.4	889	889/757/595/271
G	Cyanidin	Cy	24.6	287	287
H	Cyanidin-3-*O*-caffeoylrutinoside	Cy3CaRu	25.9	757	757/595/287
I	Cyanidin-3-*O*-(*p*-coumaroyl)glucoside-5-*O*-malonylglucoside	Cy3*p*CG5MaG	26.4	843	843/757/287
J	Pelargonidin-3-*O*-sinapylglucoside-5-*O*-glucoside	Pg3SiG5G	27.3	801	801/595/433/271
K	Pelargonidin-3-*O*-sinapylglucoside-5-*O*-arabinoside	Pg3SiG5Ara	28.0	771	771/565/433/271
L	Pelargonidin-3-*O*-ferulylglucoside-5-*O*-arabinoside	Pg3FeG5Ara	29.6	741	741/565/433/271
M	Unknown compound	UK13	30.7	-	-
N	Unknown compound	UK14	32.5	-	-

^a^ Refer to Figure 1.

## Supplementary Materials

Supplementary materials are available online.
