# Supplementary material for: Anthocyanin Profiles in Flowers of Grape Hyacinth
Source: Molecules. 2017 Apr 26;22(5):688. doi: 10.3390/molecules22050688 (PMC6154549; doi:10.3390/molecules22050688)
Supplement: Supplementary file 1 [file molecules-22-00688-s001.zip › Supplementary material-clean.pdf]

Supplementary material

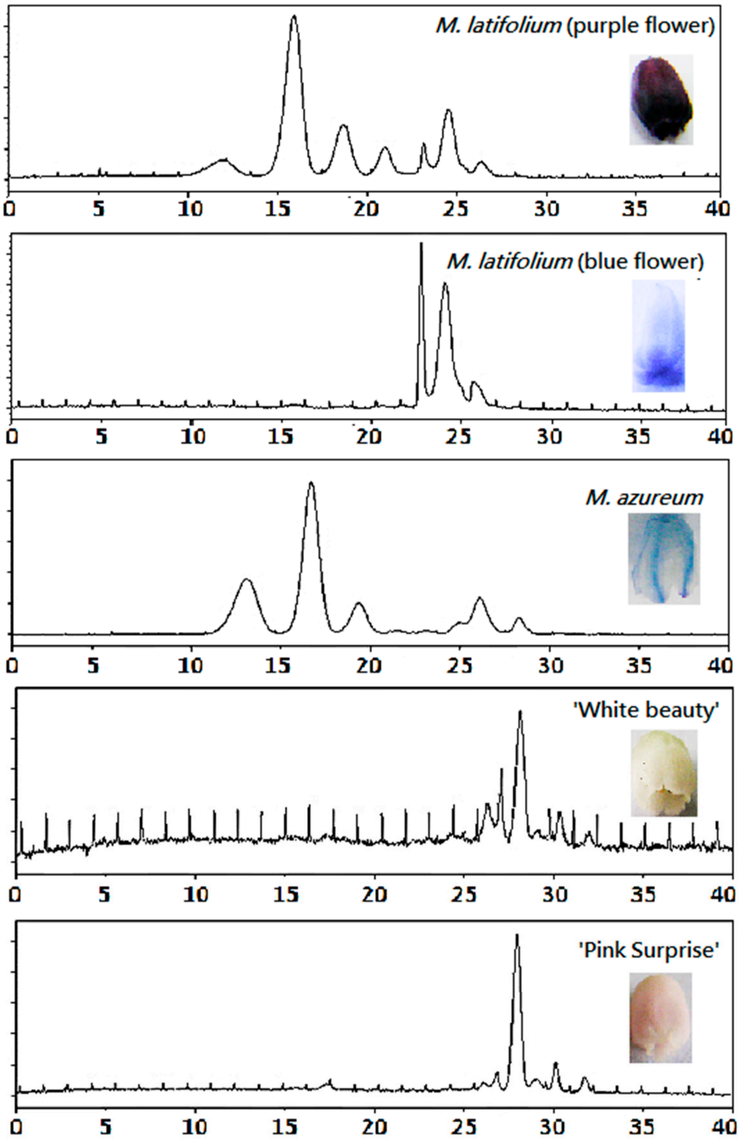

Figure S1. HPLC chromatograms of five representative samples of the main color groups.

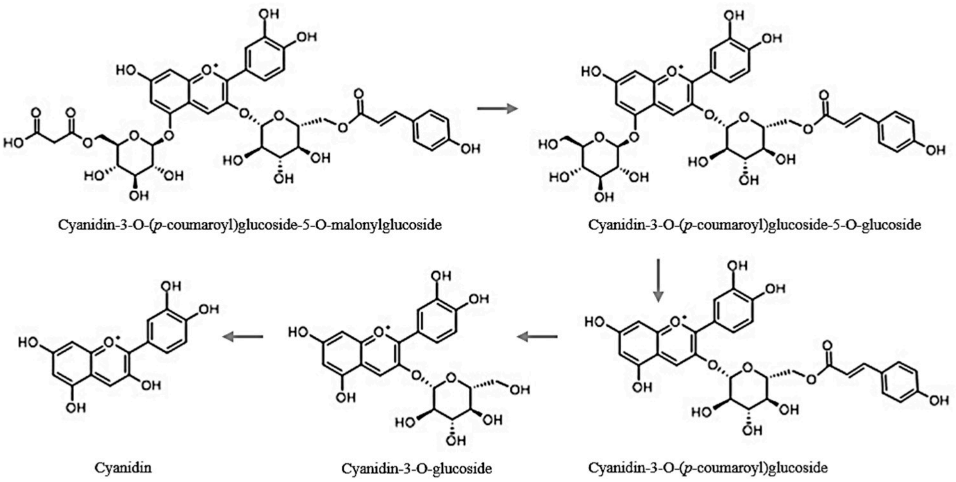

Figure S2. An illustrative example facing in detail the structural elucidation of Cyanidin-3-O-(p-coumaroyl)glucoside-5-O-malonylglucoside.

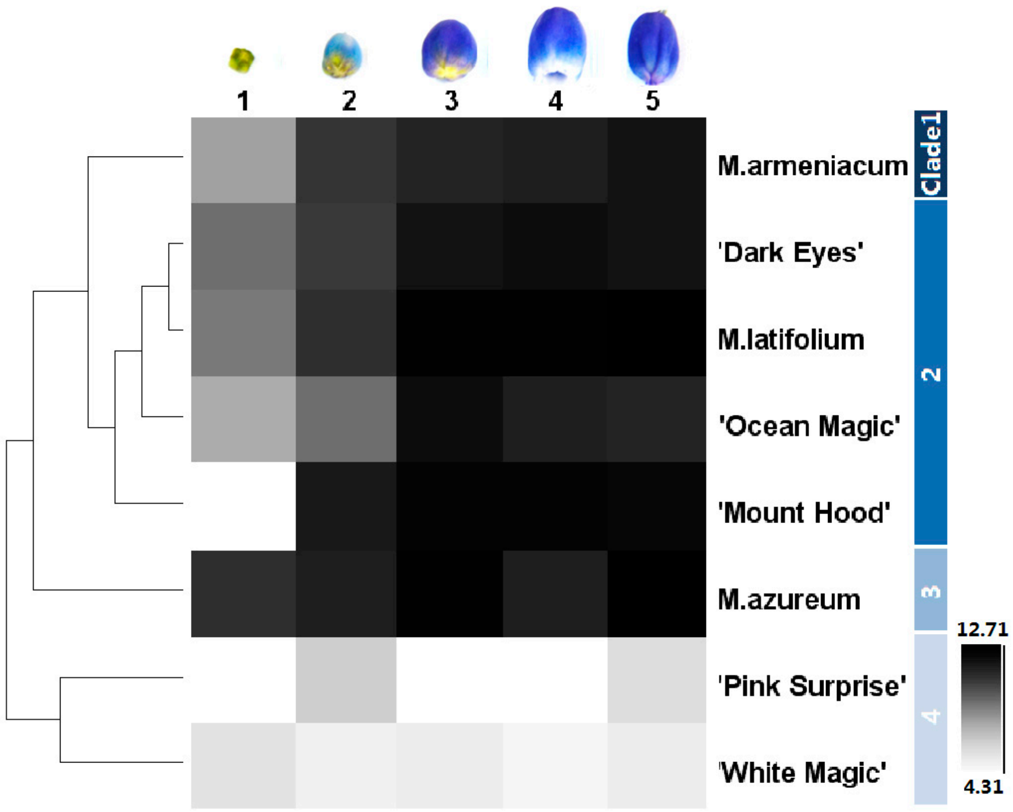

Figure S3. Changes in the total anthocyanin content of grape hyacinth tepals during flowering. The average total anthocyanin content of three independent replicates was shown on grids with different gray scale levels representing the relative log2 at different samples, respectively; 1-5 Schematic diagram of five flower developmental stages of grape hyacinth.

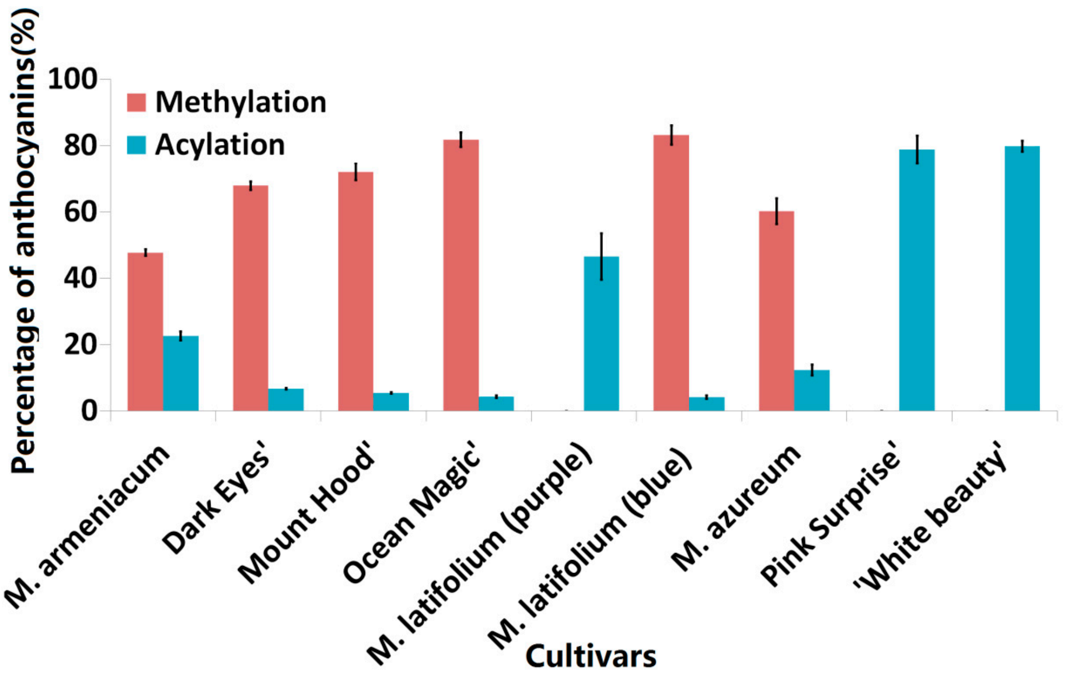

Figure S4. Percentage of methylation and acylation in anthocyanins of grape hyacinth. Percentage of methylated and acylated anthocyanins is averaged from three biological replicates. The error bars represent standard deviations.

Table S1. Color parameters of grape hyacinth varieties in different flower development stages.

Cultivars	Development Stages	Tepal Color	C*	a*/b*	h
'White Magic'	1	Greyed-green 191B	18.13±1.42	0.90	131.97±0.36
	2	Yellow-green 149C	46.42±16.49	0.61	121.39±1.53
	3	Green-white 157 D	13.86±1.46	0.46	114.57±0.59
	4	White NN155C	3.70±1.88	0.40	113.08±3.98
	5	White NN155BC	6.27±3.61	0.35	110.79±4.08
'Mount Hood'	2	Violet-blue 97BC	17.27±4.64	0.25	284.36±0.27
	3	Violet-blue 97BC	26.70±5.86	0.33	287.81±3.43
	4	Violet-blue 97A	32.52±6.26	0.23	282.70±1.10
	5	Violet-blue 98BC	46.30±5.64	0.31	287.18±2.81
'Ocean Magic'	1	Greyed Green 194BC	30.33±3.99	0.62	121.87±0.41
	2	Greyed-green 191B	18.25±1.34	0.90	132.00±0.40
	3	Violet-blue 98CD	18.04±0.92	0.03	272.06±0.34
	4	Violet-blue 95C	38.21±4.36	0.41	292.30±0.55
	5	Violet-blue 95 B	42.00±4.35	0.51	296.79±0.17
M. armeniacum	1	Green 137 ABC	23.40±2.28	0.99	134.85±2.61
	2	Violet-blue 98BC	27.90±3.25	0.17	279.49±3.21
	3	Violet-blue 95AB	42.29±5.57	0.51	296.84±0.19
	4	Violet-blue 95C	38.28±10.19	0.39	290.74±3.50
	5	Violet-blue 95AB	40.36±4.54	0.47	295.41±2.38
'Dark Eyes'	1	Greyed-white 156 B	11.30±0.61	0.23	102.99±0.79
	2	Greyed-green 191B	17.63±2.38	0.91	132.47±1.40
	3	Violet-blue 95 B	42.15±5.25	0.51	296.82±0.14
	4	Violet-blue 98C	39.28±9.91	0.27	283.80±3.49
	5	Violet-blue 96 A	42.93±2.41	0.54	298.48±1.39
M. 'Pink Surprise'	2	Red-purple 69 B	9.35±1.94	33.33	358.40±0.49
	3	Red-purple 65 D	11.28±0.02	4.55	12.61±0.11
	4	Red-purple 62C	25.58±0.02	25.00	2.27±0.68
	5	Red-purple 65AB	33.64±0.02	100.00	0.42±0.03
M. latifolium (upper flower)	4	Violet-blue 93 B	28.32±1.06	2.13	297.72±0.18
M. latifolium (lower flower)	2	Purple-violet N82 D	25.92±0.02	1.20	320.27±0.03
	3	Purple 79 B	19.45±0.04	1.79	330.58±0.04
	4	Purple 79 B	31.16±3.02	1.79	343.09±10.81
	5	Purple N79 A	47.59±1.01	3.13	349.47±0.24
M. azureum	1	Green 138AB	17.42±1.43	0.89	132.2±0.43
	2	Violet-blue 98BCD	8.57±1.52	1.10	131.64±0.81
	3	Violet-blue 94AB	27.74±3.21	0.17	137.80±7.70
	4	Violet-blue 96BC	17.06±4.66	0.24	279.13±1.30
	5	Violet-blue 96AB	36.97±7.43	0.53	283.39±3.25

Note： Flower colour was determined using RHSCC and recorded as three dimensional CIEL*a*b* values. Flower colour was reproduced by an image editing software (Photoshop) using mean L, a*, b* values at all development stages investigated in this study. Chroma C* represents the brightness of the colour. a*/b*represents the hue. h means hue angle. The values are mean ±SD (n=5).
